# Identification of Key Transcription Factors and Immune Infiltration Patterns Associated With Breast Cancer Prognosis Using WGCNA and Cox Regression Analysis

**DOI:** 10.3389/fonc.2021.742792

**Published:** 2021-12-21

**Authors:** Xin Yin, Jiaxiang Liu, Xin Wang, Tianshu Yang, Gen Li, Yaxin Shang, Xu Teng, Hefen Yu, Shuang Wang, Wei Huang

**Affiliations:** ^1^ Beijing Key Laboratory of Cancer Invasion and Metastasis Research, Department of Biochemistry and Molecular Biology, School of Basic Medical Sciences, Capital Medical University, Beijing, China; ^2^ Department of Breast Surgical Oncology, National Cancer Center/National Clinical Research Center for Cancer/Cancer Hospital, Chinese Academy of Medical Sciences and Peking Union Medical College, Beijing, China; ^3^ Department of Cardio Surgery Center, Shandong Second Provincial General Hospital, Jinan, China

**Keywords:** transcription factors, WGCNA, COX regression analysis, LASSO analysis, immune infiltration, breast cancer

## Abstract

Breast cancer is the most frequently diagnosed cancer and the second leading cause of cancer death among women worldwide. Therefore, the need for effective breast cancer treatment is urgent. Transcription factors (TFs) directly participate in gene transcription, and their dysregulation plays a key role in breast cancer. Our study identified 459 differentially expressed TFs between tumor and normal samples from The Cancer Genome Atlas database. Based on gene expression analysis and weighted gene co-expression network analysis, the co-expression yellow module was found to be integral for breast cancer progression. A total of 121 genes in the yellow module were used for function enrichment. To further confirm prognosis-related TFs, COX regression and LASSO analyses were performed; consequently, a prognostic risk model was constructed, and its validity was verified. Ten prognosis-related TFs were identified according to their expression profile, survival probability, and target genes. COPS5, HDAC2, and NONO were recognized as hub TFs in breast cancer. These TFs were highly expressed in human breast cancer cell lines and clinical breast cancer samples; this result was consistent with the information from multiple databases. Immune infiltration analysis revealed that the proportions of resting dendritic and mast cells were greater in the low-risk group than those in the high-risk group. Thus, in this study, we identified three hub biomarkers related to breast cancer prognosis. The results provide a framework for the co-expression of TF modules and immune infiltration in breast cancer.

## Introduction

Transcription factors (TFs) identify specific DNA promoters to control chromatin and transcription in the process from gene to protein (1). TFs are spatially, temporally, and sequentially expressed in tissues during cell development, proliferation, or differentiation processes; and any modification of their expression and functional disorder may result in master deregulation of cell integrity or organism homeostasis leading to pathologies. TFs are able to activate or repress gene transcription depending on the specific structure of their DNA-binding domain, including structural motifs, such as the C_2_H_2_ homeodomain, helix–loop–helix, helix–turn–helix, and leucine zipper (2). Similarly, the expression of TFs is tissue- and cell-type-specific and often indicative of corresponding specific functions (3). Numerous diseases arise from a breakdown in the regulation of TFs: a third of human developmental disorders have been attributed to dysfunctional TFs, and a majority of oncogenes are also TFs (4–6). In recent years, an increasing number of studies have found that drugs targeting TFs can modulate some hallmark properties of cancer. For example, MLL-AF9 is a driver of the leukemia stem cell population (7); GABP increases expression of TERT in glioblastomas with a mutant TERT promoter (8); PML–RARα blocks cell differentiation in acute promyelocytic leukemia (9); RUNX1–ETO reduces CD48, thereby decreasing NK cell killing (10); Runx2 and SIX1 induce epithelial-mesenchymal transition (EMT) and breast cell invasion (11, 12).

Breast cancer is the most frequently diagnosed cancer and is the leading cause of cancer death among women worldwide (13). Breast cancer is a heterogeneous disease, which is a result of genetic alteration or epigenetic modifications by multiple factors, especially the TFs. TFs are divided into three groups in breast cancer: steroid receptors, resident nuclear TFs, and latent cytoplasmic factors (14). These TFs regulate cell cycle, stem cell differentiation, apoptosis, migration, and cell differentiation and modulate breast cancer progression. In our previous study, we demonstrated that the TF, GATA3, could recruit histone demethylase UTX to suppress metastasis of breast cancer (15). In addition, the TF, RUNX2, can promote CD44/CD24 breast cancer stem cell properties and breast cancer tumorigenesis through the EMT process (16). Furthermore, the TF, GATA4, induces cell cycle arrest and apoptosis through NF-κB signaling in breast cancer cells (17). To date, there are approximately 1508 TF genes that participate in sequencer-specific DNA binding (2); however, their roles in breast cancer have not been elucidated.

The availability of complete genome sequences and the development of high-throughput sequencing techniques have provided complementary information describing the function of TFs in cancer. Weighted gene co-expression network analysis (WGCNA) is a new bioinformatics method, which allows for a comprehensive interpretation of gene expression data by constructing gene networks based on similarities in expression profiles among samples based on microarray or RNA-seq data (18). Closely connected genes that have been proven to be conserved across phylogenies and enriched in protein-protein interactions are grouped into one module (19). WGCNA is the most widely used co-expression network technique and is often used in genetic analysis of cancer (20). By constructing two gene networks according to normal and tumor tissue gene expression data, it is possible to identify critical modules and genes that might be involved in pathological processes and, subsequently, are able to serve as diagnostic or prognostic biomarkers, or potential therapeutic targets. In breast cancer, the novel microRNA biomarkers for each subtype of breast cancer can be detected using WGCNA (21). In addition, our previous study identified one lncRNA and five mRNA that serve as important prognostic biomarkers in breast cancer (22).

In this study, we used WGCNA to compare the TF expression of a patient with breast cancer and that of a normal patient, based on information from The Cancer Genome Atlas (TCGA), and identified the most significant modules related to breast cancer. More importantly, we also used the Least Absolute Shrinkage and Selection Operator (LASSO) to construct a TF–target network and identify 10 key TFs related to patient survival and immune infiltration in breast cancer. Upon further analysis, three hub TFs, COPS5, HDAC2, and NONO were identified and were demonstrated to be highly expressed in human breast cancer samples when compared to adjacent tissues using western blotting and quantitative reverse transcription polymerase chain reaction (qRT-PCR). Our study not only detected 10 TFs correlated with breast cancer prognosis, but also provides a direct reference for exploring the roles of TFs and target genes in breast cancer.

## Results

### Identification of Differentially Expressed TFs and Subsequent Gene Function Enrichment Analysis

**Figure 1:**
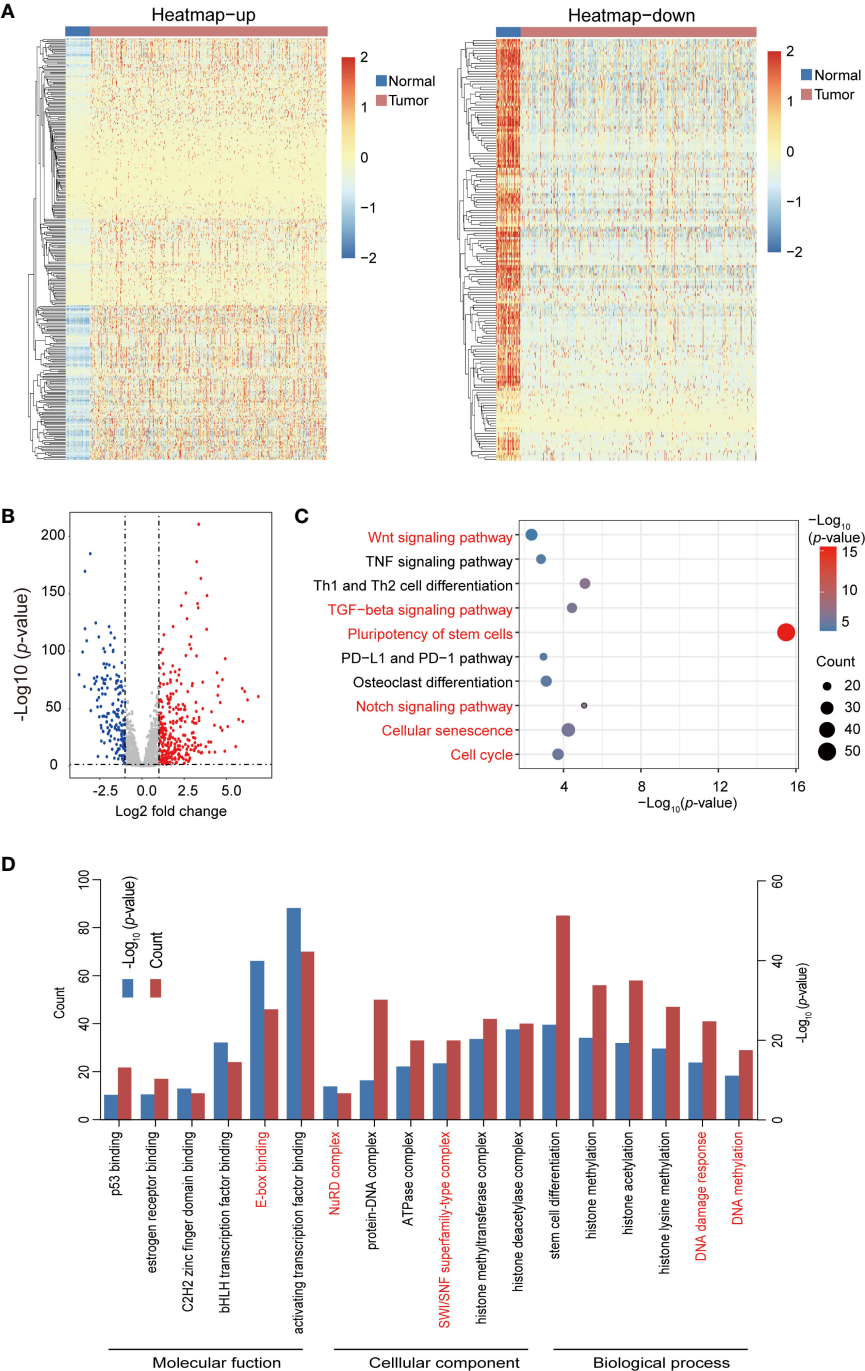
Heatmap, volcano plots, and gene enrichment analyses of differential transcription factors (TFs). **(A)** Heatmap of differential TFs. **(B)** Volcano plot of differential TFs. **(C)** Kyoto Encyclopedia of Genes and Genomes (KEGG) pathway enrichment of differential TFs. Red pathways are common between the total differential TFs of the yellow module. **(D)** Gene ontology (GO) enrichment of total differential TFs.

JASPAR, TRANSFAC, CISBP, and TRRUST databases were used for the bioinformatics analysis process. A total of 1930 TFs were obtained after the removal of duplicated TFs from the four databases, as shown in **Table 1**. The raw counts data and clinical data (**Table 3**) of breast cancer were download from TCGA. After the raw data were statistically analyzed using the DESeq2 R package, 295 significantly upregulated and 164 significantly downregulated TFs were screened out (**Supplemental Table 1**). A volcano plot and heatmaps were generated to demonstrate the distribution of 459 differentially expressed TFs (**Figures 1A, B**). In order to further analyze the function of these TFs, the “clusterProfiler” R package was used to conduct the Gene ontology (GO) and Kyoto Encyclopedia of Genes and Genomes (KEGG) pathway annotation analyses for the 459 differential TFs (**Supplemental Table 2**). Enrichment results were visualized using the “ggplot2” R package (**Figures 1C, D**). Signaling pathways found to regulate the pluripotency of stem cells, Th1 and Th2 cell differentiation, the Notch signaling pathway, the TGF-beta signaling pathway, cellular senescence, and the cell cycle, among others, were obtained from the KEGG pathway enrichment analysis (**Figure 1C**). Moreover, GO analysis showed that these TFs were significantly enriched in histone methylation, histone acetylation, DNA damage response, stem cell differentiation, and TF binding activation (**Figure 1D**).

**Table 1:** TFs database information.

Database	CISBP	TRRUST	JARSPAR	TRANSFAC	Total
TFs	1639	795	111	287	1930

### Construction of Co-Expression Modules of Differentially Expressed TFs Using WGCNA

**Figure 2:**
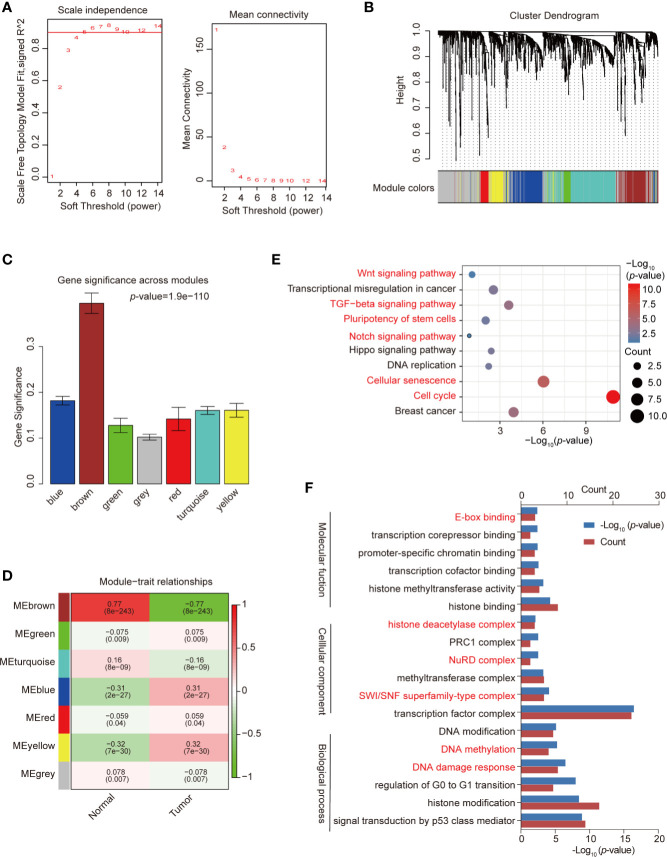
Construction of co-expression modules by weighted gene co-expression network analysis (WGCNA). **(A)** Analysis of network topology for various soft-threshold powers. Check scale-free topology; the adjacency matrix was defined using soft-thresholds with β = 4. **(B)** Clustering dendrograms of transcription factors (TFs), with dissimilarity based on topological overlap, together with assigned module colors. **(C)** Bar plot of mean significance across modules; gene significance represented the correlation between module and breast cancer. **(D)** Analysis of module-breast cancer relationships. **(E)** Kyoto Encyclopedia of Genes and Genomes (KEGG) pathway enrichment of TFs in the yellow module. **(F)** Gene ontology (GO) enrichment of differential TFs in the yellow module. Red pathways are common with total differential TFs.

WGCNA was used to construct a co-expression network and modules of differentially expressed TFs exhibiting a strong correlation with breast cancer. The Pearson’s correlation matrix of the genes was converted into a strengthening adjacency matrix by power β = 4 based on a scale-free topology with R^2^ = 0.97 (**Figure 2A**). All of the TFs were clustered using a topological overlap matrix (TOM)-based dissimilarity measure according to the Dynamic Tree Cut algorithm to divide the tree into eight modules (**Figures 2B, C**) labeled with different colors. Then, we summarized the gene co-expression by eigengenes and calculated the correlation of each eigengene with breast cancer. The association between co-expression modules and breast cancer is shown in **Figure 2D**. The yellow module exhibited a strong positive relationship with breast cancer and was used for further analysis. The TFs in the yellow module are listed in **Supplemental Table 3**.

We used the “clusterProfiler” R package to analyze the GO and KEGG pathway enrichment of the 121 TFs in the co-expression yellow module (**Supplemental Table 4**). The significant enrichment function and pathways (*p-*value < 0.05) are shown in **Figures 2E, F**. KEGG signaling pathways possibly related to breast cancer progression were identified (**Figure 2E**), including the cell cycle, cellular senescence, the TGF-β signaling pathway, and the Hippo signaling pathway. GO data revealed that the TFs were enriched in pathways such as DNA damage response, DNA methylation, TF complex, SWI/SNF superfamily-type complex, and histone binding (**Figure 2F**), which represent the classical function of TFs.

### Construction of Prognostic TFs of Breast Cancer

**Figure 3:**
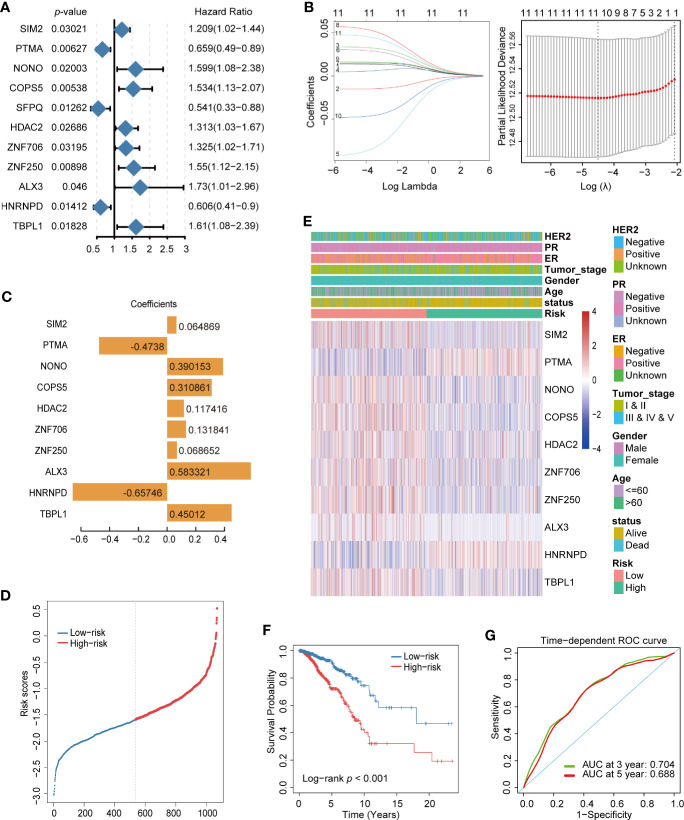
Construction of prognostic transcription factor (TF) model of breast cancer. **(A)** Single variable Cox regression analysis of differential TFs. **(B)** Line plot of Least Absolute Shrinkage and Selection Operator (LASSO) regression analysis of 11 TFs in breast cancer. **(C)** LASSO model (λ) of breast cancer. **(C)** Coefficient spectrum of the 10 prognosis-related TFs. **(D)** Distribution of risk score in breast cancer. **(E)** Heatmap of the 10 prognosis-related TF expression profiles combined with clinical traits in the high- and low-risk groups. **(F)** Kaplan–Meier analysis of high- and low-risk groups for breast cancer from TCGA. **(G)** ROC curve of 3- and 5- year survival probability in breast cancer from TCGA.

In order to determine the key TFs related to the prognosis of breast cancer, TFs significantly related to cancer prognosis were identified by single variable Cox regression analysis. As shown in **Figure 3A**, 11 TFs were obtained with *p*-values <0.05. Next, LASSO regression analysis was used to remove redundant TFs to get the prognostic TFs based on the result of single variable Cox regression analysis (**Figure 3B**). One TF was abandoned, and the remaining 10 TFs further analyzed were SIM2, PTMA, NONO, COPS5, HDAC2, ZNF706, ZNF250, ALX3, HNRNPD, and TBPL1. They were analyzed using multivariate Cox regression analysis and utilized to construct a final prognostic model associated with TFs of breast cancer. The coefficients of each TF are shown in **Figure 3C**. The prognosis of breast cancer was evaluated by calculating the risk score of each patient on the basis of the expression and coefficients of each TF. breast cancer patients were divided into high-risk and low-risk groups according to their risk score (**Figure 3D**). A heatmap was generated to directly present the relationship between the differential genes of high-risk and low-risk groups and the related traits (**Figure 3E**). Furthermore, to test the effectiveness of the prognostic model, a breast cancer dataset, Metabric, from the Cbioportal database was used as a training set and plotted using a Kaplan–Meier analysis and receiver operating characteristic (ROC) curve of 3- and 5-year survival probability. The survival time of patients with a low-risk score was significantly longer than that of patients with a high-risk score in both models (**Figure 3F** and **Supplemental Figure 1A**), and the area under curve of the model indicated that it had the ability to predict the prognosis of breast cancer (**Figure 3G** and **Supplemental Figure 1B**).

**Figure 4:**
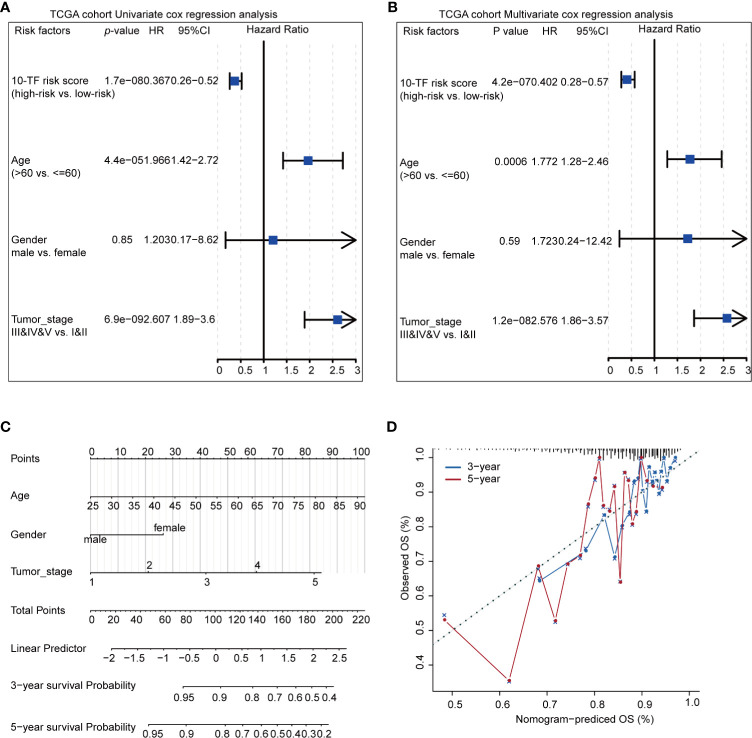
Prediction of the prognosis probability in breast cancer. **(A)** Single variable Cox regression analysis in breast cancer cohort. **(B)** Multivariate Cox regression analysis in breast cancer cohort. **(C)** A nomogram of the breast cancer cohort. **(D)** Calibration maps of the breast cancer cohort.

Finally, we assessed the prognosis value of ten TFs based on risk score, age, gender, and tumor stage. According to univariate cox regression analysis, the risk score was significantly correlated with overall survival (OS) (HR = 0.367, 95% CI = 0.26–0.52, *p-*value < 1.7e-08) (**Figure 4A**); meanwhile, according to multivariate Cox regression analysis, the risk score was an independent prognostic indicator (HR = 0.402, 95% CI = 0.28–0.57, *p-*value < 4.2e-07) (**Figure 4B**). Nomogram and calibration maps were used to quantify the contribution of individual factors in the clinical prognosis and verify the validity of this model, respectively (**Figures 4C, D**). The results indicated that this prognostic model had good predictive capabilities.

### Analysis of Immune Infiltration

**Figure 5:**
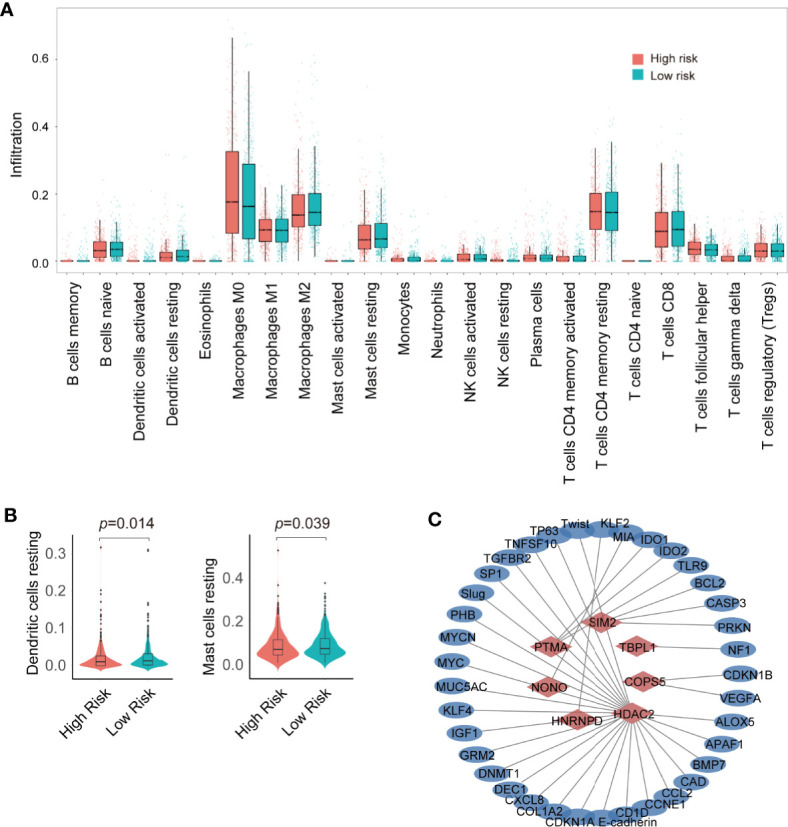
Analysis of immune infiltration and construction of transcription factor (TF)-target gene network. **(A)** Analysis of immune infiltration in the high- and low-risk groups. **(B)** violin plot of resting dendritic and mast cells in the high- and low- risk groups. **(C)** Seven TF–target gene network.

The tumor microenvironment plays a crucial role in tumor progression, therapeutic response, and patient outcomes. Immune-infiltrating cells are an important component of the tumor microenvironment (23). To detect the difference in the immune infiltration status of the high-risk and low-risk groups, CIBERSORT was used to screen 22 immune cell types, including T cells, B cells, macrophages, natural killer cells, eosinophils, neutrophils, and dendritic cells, for significant immune infiltration-related cells. There were 22 kinds of immune infiltration-related cells that were different between the high-risk and low-risk groups (**Figure 5A**). Resting dendritic and resting mast cells were the most significant immune infiltration-related cells and were highly expressed in low-risk group (**Figure 5B**).

### Construction of the TF–Target Gene Network and Expression Profile of 10 TFs

**Figure 6:**
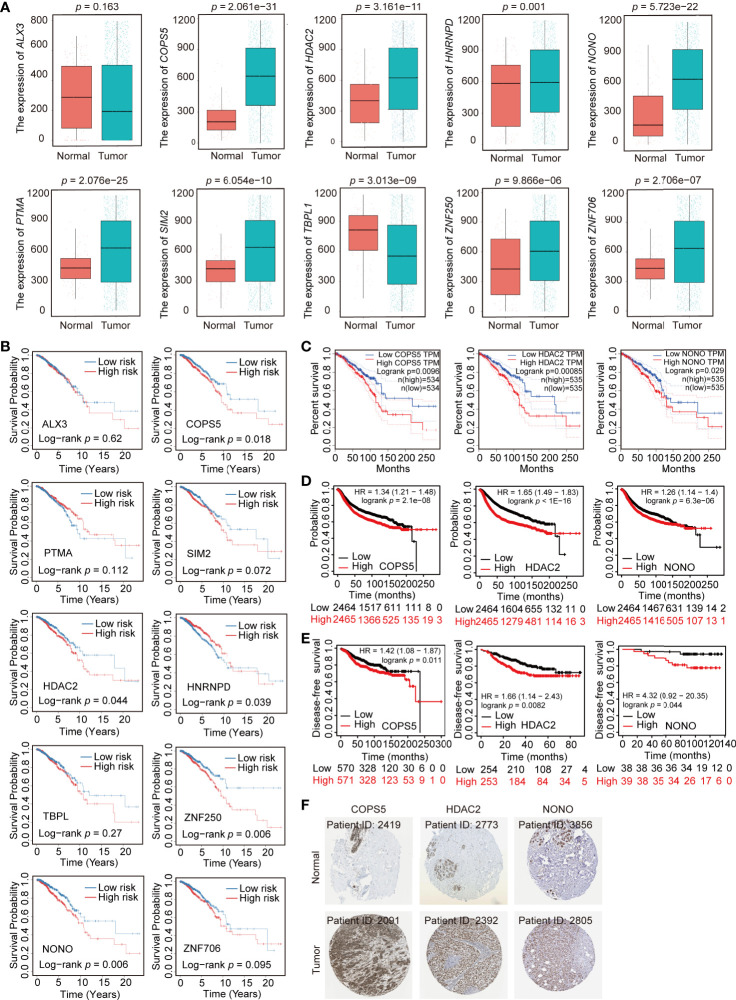
Expression profile and survival analysis of 10 transcription factors (TFs) in breast cancer. **(A)** Expression profile of 10 TFs (ALX3, COPS5, HDAC2, HNRNPD, NONO, PTMA, SIM2, TBPL1, ZNF250, and ZNF706) in normal and breast cancer samples. **(B)** Kaplan–Meier analysis of 10 TFs (ALX3, COPS5, HDAC2, HNRNPD, NONO, PTMA, SIM2, TBPL1, ZNF250, and ZNF706) in breast cancer. **(C, D)**. Association of COPS5, HDAC2, and NONO with overall survival in the online database (http://gepia.cancer-pku.cn/ and https://kmplot.com/analysis/). **(E)** Association of COPS5, HDAC2, and NONO with disease-free survival in the online database (https://kmplot.com/analysis/). **(F)** Immunohistochemistry (IHC) of COPS5 (COPS5 in normal sample from 2419; COPS5 in breast cancer sample from 2091), HDAC2 (HDAC2 in normal sample from 2773; HDAC2 in breast cancer sample from 2392), and NONO (NONO in normal sample from 3856; NONO in breast cancer sample from 2805) in breast cancer and normal samples from the HPA database (https://www.proteinatlas.org/).

TFs generally regulate gene expression by binding with the promoter of target genes. To predict the target genes of 10 prognostic TFs, the TRRUST database was used to construct a TF regulatory network (**Supplemental Table 5**). HDAC2 had 28 target genes; PTMA had 4 target genes; SIM2 had 3 target genes; COPS5 had 2 target genes; and HNRNPD, NONO, and TBPL1 had one target gene (**Figure 5C**). However, ZNF706, ZNF250, ALX3 had no target genes. In addition, we compared the expression profile of these 10 TFs in normal and breast cancer tissue obtained from TCGA. The expression of COPS5, HDAC2, HNRNPD, NONO, PTMA, SIM2, ZNF250, and ZNF706 was significantly higher in breast cancer tissue than that in normal tissue, whereas ALX3 and TBPL1 exhibited higher expression in normal tissue than in breast cancer tissue (**Figure 6A**). Moreover, Kaplan–Meier analysis of each TF indicated that COPS5, HDAC2, NONO, and ZNF250 are associated with a lower survival probability in the high-risk group than in the low-risk group (**Figure 6B**). Results of the TF–target gene network, expression panel, and survival analysis revealed that COPS5, HDAC2, and NONO served as the hub TFs for breast cancer. Moreover, these three TFs were verified in other databases. High expression of COPS5, HDAC2, and NONO was associated with poor OS and disease-free survival in patients with breast cancer (**Figures 6C–E**) (http://gepia.cancer-pku.cn/ and https://kmplot.com/analysis/), and the associated protein expression was also high in breast cancer (**Figure 6F**) (https://www.proteinatlas.org/). Altogether, these data suggested that COPS5, HDAC2, and NONO were related to poor prognosis.

### Validation of the Hub TF mRNA and Protein Expression Using qRT-PCR and Western Blotting

**Figure 7:**
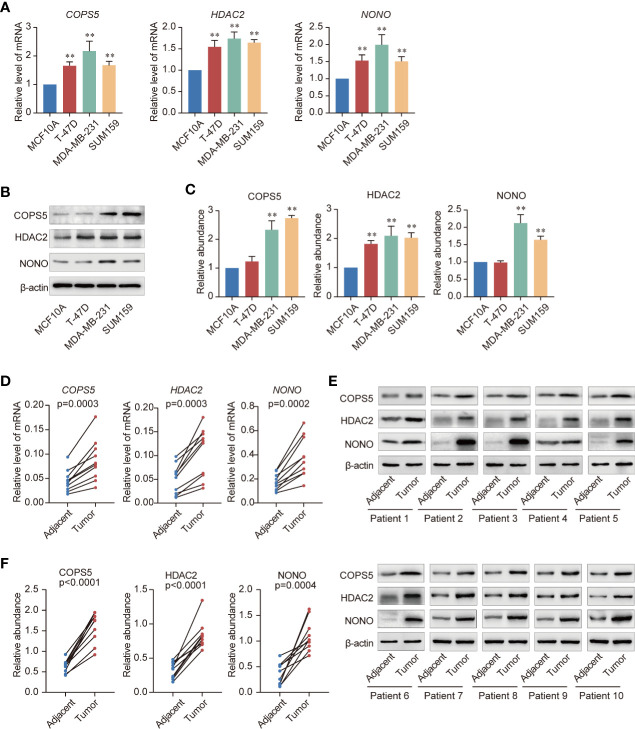
Validation of three key genes. **(A)** Expression of *COPS5*, *HDAC2*, and *NONO* mRNAs in the normal breast epithelial cell line MCF10A and the triple-negative breast cancer cell lines T-47D, MDA-MB-231, and SUM159 evaluated using quantitative reverse transcription polymerase chain reaction (qRT-PCR). **(B)** Expression of COPS5, HDAC2, and NONO the normal breast epithelial cell line MCF10A and the triple-negative breast cancer cell lines T-47D, MDA-MB-231, and SUM159 analyzed using western blotting. **(C)** Gray scanning of the protein expression results in **(B, D)**. Analysis of expression of *COPS5*, *HDAC2*, and *NONO* mRNAs in adjacent and tumor tissues of breast cancer samples using qRT-PCR (n = 10 in each group). **(E)**. Analysis of expression of COPS5, HDAC2, and NONO proteins in adjacent and tumor tissues of breast cancer samples using western blotting (n = 10 in each group). **(F)** Gray scanning of the protein expression results in **(E)**. Error bars represent mean ± SD of three independent experiments. *p < 0.05, **p < 0.01. Student’s t-test.

To further confirm whether COPS5, HDAC2, and NONO performed similar functions in normal and breast cancer cells, we measured the mRNA and protein levels in breast cancer and normal breast epithelial cells. Consistent with the bioinformatics analysis, COPS5, HDAC2, and NONO were more highly expressed in breast cancer cell lines than in normal breast epithelial cells (**Figures 7A, B**). Furthermore, we collected tumor and adjacent tissues from 10 breast cancer patients from the National Cancer Center of China and analyzed the mRNA and protein expression in tumor and adjacent tissues. The results showed that both mRNA and protein levels were significantly higher in tumor tissues than in adjacent tissues (**Figures 7C–F**), which is consistent with the results of the TCGA breast cancer cohort analysis.

## Discussion

TFs are involved in various human diseases, such as cancers, for which they account for about 20% of all oncogenes identified so far. Currently, studies have shown that TFs might be as new therapy target for cancer in clinical settings by modulating their expression or degradation, blocking protein/protein interactions, or targeting the TF itself to prevent its DNA binding either through a binding pocket or at the DNA-interacting site (4, 24, 25). It is urgent to screen out critical TFs and explore their mechanism in cancers to estimate whether they could be a potential therapy target. In our study, we present for the first time, to our knowledge, the identification of prognosis-related TFs in breast cancer using WGCNA and COX regression analysis. Briefly, WGCNA was used to identify biologically meaningful modules within the key networks involving functionally related TFs in breast cancer. Multiple co-expression modules were constructed combined with breast cancer processing. The yellow module was selected for further analysis because of its strong positive correlation with breast cancer. In order to screened for prognostic TFs, univariate and multivariate COX regression analyses were used to construct and verify the breast cancer prognosis model. Ten prognostic TFs (SIM2, PTMA, NONO, COPS5, HDAC2, ZNF706, ZNF250, ALX3, HNRNPD, and TBPL1) were identified by LASSO regression analysis. Meanwhile, we also analyzed the immune infiltration in the high- and low-risk groups, the results of which demonstrated that resting dendritic and mast cells were significantly higher in the low-risk group. To further isolate the hub TFs from these 10 TFs, we constructed a TF–target gene network, and statistically analyzed the expression level of the 10 TFs in normal and breast cancer samples and determined the survival probabilities for the high- and low-risk groups. Finally, COPS5, HDAC2, and NONO were selected as hub TFs because of their significantly higher expression in breast cancer, lower survival probability in the high-risk group, and specific target genes. We combined WGCNA and COX regression analysis to establish the independent prognostic factor as well as validity of the prognostic model and validated the three hub genes COPS5, HDAC2, and NONO as breast cancer prognosis factors.

As one of the hub genes of breast cancer prognosis, COPS5, also known as JAB1 or CSN5, was initially identified as c-Jun activation domain-binding protein-1 and is aberrantly overexpressed in various human cancers including breast cancer. Previous studies have demonstrated that COPS5 is involved in controlling cell proliferation, cell cycle, apoptosis, DNA damage response, and drug resistance, which are critical processes in tumorigenesis (26–29). The TF–target gene network showed that the target genes of COPS5 in breast cancer were *CDKN1B* and *VEGFA* (**Figure 5C**), which were suggested to block G1 growth induced by COPS5 in MDA-MB-453 cells (30), promote breast cancer progress *via* the HIF1A signaling pathway, and degrade p53 (31). In this regard, we speculate that COPS5 may regulate VEGFA to promote breast cancer progression, although the mechanism between VEGFA and COPS5 needs to be further explored. Furthermore, COPS5 was found to be involved in breast cancer metastasis as a target gene of miRNA let-7d (32). HDAC2 is an important histone deacetylase that is overexpressed in breast cancer. According to the TF–target gene network, HDAC2 might participate in various cellular processes, such as the EMT (target genes: E-cadherin, Slug, Twist), immunoreaction (target genes: ALOX5, CCL2, CD1D, CXCL8, SP1, TGFBR2), and cell stem (target genes: KLF4, MYC) (**Figure 5C**). HDAC2 can be recruited by PELP1 to miR-200a and miR-141 promoters and suppress their expression to promote EMT in breast cancer (33). Another study also revealed that the Snail/HDAC1/2 complex was recruited by SREBP1 to repress E-cadherin expression in breast cancer (34). Moreover, HDAC2 transcription is promoted by the YAP/RUNX1 complex to induce chemoresistance and stemness in breast cancer, indicating that HDAC2 plays a role in cell stem progress (35). Consistent with our results, HDAC2 might be involved in the immune response by regulating the expression of proinflammatory genes following stimulation with LPS (36). Moreover, HDAC2 also promoted IFNγ-induced PD-L1 expression to enhance antitumor immunity and tumor proliferation and metastasis (37). More importantly, our recent study demonstrated that the COPS5-associated protein CUL4B could interact with HDAC-containing complexes to promote EMT and stem cell production in breast cancer (38). Additionally, we reported that an epigenetic small-molecule inhibitor of HDACs, PCI-24781, could target RGS2 to reduce cell proliferation, metastasis, and differentiation, resulting in cell death during breast cancer progression (39). Thus, HDACs might serve as a potential therapeutic target for breast cancer. Apparently, NONO is also a prognostic factor of breast cancer. Several studies have indicated that NONO is a factor that controls DNA damage (40), cell proliferation (41), metabolism (42), and drug resistance (43, 44) in breast cancer. In addition, NONO, a nuclear protein, could interact with MSN to phosphorylate CREB and upregulate downstream gene expression as well as promote the progression of breast cancer (44). Furthermore, NONO promoted breast cancer metastasis through induction by lncRNA T-UCRs (45). Thus, we observed that these three hub genes acted as pro-metastatic and prognostic factors in breast cancer; however, the specific mechanism behind this discrepancy requires further investigation.

In the breast tumor microenvironment, intimate mixtures of cancer cells and non-cancer cells subsist. Recent drug trials that target immune checkpoints indicate that infiltrating immune cells, not cancer cells, seems the most likely to improve clinical outcomes and to be effectively targeted by drugs (46, 47). According to the results of our CIBERSORT analysis, the number of resting dendritic and mast cells was significantly higher in the low-risk group. Dendritic cells are professional antigen presenting cells that function as the basis of the adaptive immune response. As antigen-presenting cells, dendritic cells help shape the adaptive immune response (23). Therefore, dendritic cells are being extensively evaluated for their clinical potential as an anticancer immunotherapeutic cell product to induce and/or enhance tumor-specific immune response (48). Mast cells are tissue-resident, innate immune cells that play a key role in the inflammatory response and tissue homeostasis. These cells accumulate in the tumor stroma of different human cancer types, and their increased density has been associated with either good or poor prognosis, depending on the tumor type and stage (49). Mast cells are immune cells present in all classes of vertebrates, which have the capacity to rapidly perceive metabolic and immunologic insults and initiate different biochemical programs of homeostasis or inflammation (50). These cells are recruited into the tumor microenvironment by several tumor cell-derived chemotactic factors such as VEGFs and ANGPT1, and exert pro- or anti-tumorigenic effects depending on tumor type, model, and stage (51–53). One recent study showed that mast cells exhibit diverse functional potential in different cancer types and have higher proportions in most cancer types, supporting the assertion that mast cells accumulate in tumors and play important roles in tumorigenesis and tumor progression (54). Our results might demonstrate that resting dendritic and mast cells might play an anti-tumorigenic role in breast cancer.

In conclusion, our study used a breast cancer dataset from the TCGA database and compared differential TFs between normal and breast cancer samples. WGCNA was used to construct a free-scale network between normal and breast cancer samples to further identify the co-expression yellow module. This module was found to be significantly associated with breast cancer progression. Gene function enrichment and a prognosis-related risk model were performed to identify 10 prognosis-related TFs (SIM2, PTMA, NONO, COPS5, HDAC2, ZNF706, ZNF250, ALX3, HNRNPD, and TBPL1). Moreover, three hub TFs (COPS5, HDAC2, and NONO) were isolated as the prognostic biomarkers of breast cancer through the TF–target gene network, expression profile, and survival probability of the 10 prognosis-related TFs in breast cancer. In addition, these three TFs were verified in multiple databases and human breast cancer cell lines and samples. However, there were some inevitable limitations of our study. First, the target genes of TFs and their molecular mechanism need to be confirmed and explored *in vivo* and *in vitro*. Second, other key clinical pathological features, such as metastasis, were not included. Further studies are warranted to explore and demonstrate the molecular mechanism of breast cancer and provide convincing data for clinical treatment of breast cancer.

## Materials and Methods

### Collection of TF Dataset and Preprocessing of TCGA Data of Breast Cancer Patients

**Figure 8:**
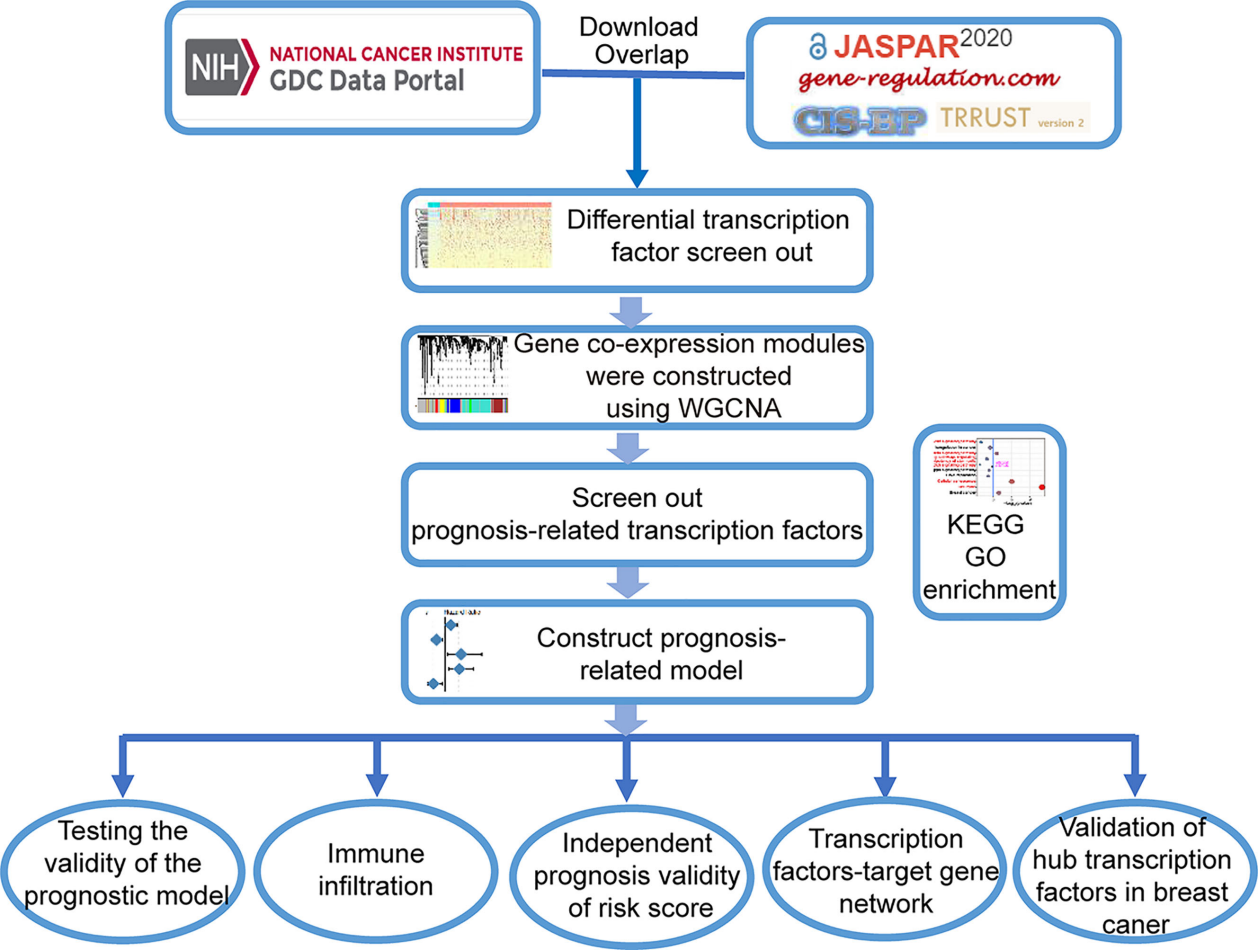
Flow chart of analyses.

The workflow of data analysis is shown in **Figure 8**. TFs from the four most common TF databases, JASPAR (http://jaspar.genereg.net/), TRANSFAC (http://gene-regulation.com/pub/databases.html), CISBP (http://cisbp.ccbr.utoronto.ca/), and TRRUST (https://www.grnpedia.org/trrust/), were overlapped and used to construct the dataset. Breast cancer-related raw RNA-seq and clinical data (**Table 2**) were downloaded from TCGA database (https://www.cancer.gov/about-nci/organization/ccg/research/structural-genomics/tcga). Differential genes were identified between the tumor and normal samples using the DESeq2 R package, at thresholds of |log2FC| >1 and adj-*p <*0.05. Thereafter, 459 differential TFs were selected for further analysis by taking the intersection of the TF dataset and the breast cancer differential genes.

**Table 2:** TCGA-breast cancer clinical Information.

Characteristic	No. of Patients
**Stage**	
I & II	797
III & IV & V	271
**Status**	
Alive	149
Dead	919
**Age**	
≤60	597
>60	471
**Gender**	
Male	12
Female	1056

### WGCNA of Differential TFs

WGCNA was run as described previously (22). Briefly, a similarity matrix was constructed using expression data and converted into an adjacency matrix, a_ij_. Next, the adjacency matrix was converted into a TOM as an input for the hierarchical clustering analysis of genes. The most representative genes, module eigengenes (MEs), were the first principal components, representing the overall level of gene expression in individual modules. Finally, the gene significance (GS) was evaluated using other biological information. The expression profile of TFs was used to construct a free-scale network and identify significant modules between normal and breast cancer-related genes to analyze differential genes in these modules.

### Gene Function Enrichment of Differential TFs

Functional and pathway enrichment of differential TFs were performed using the KOBAS 3.0 online database (http://kobas.cbi.pku.edu.cn/kobas3). Significantly enriched functions and pathways were visualized by R package ggplot2 with RStudio (Version 3.6.3).

### Construction of Cox Regression Model

Differential TF expression profiles in the yellow module were used for single variable Cox proportional risk regression analysis by using “survival” and “survminer” R packages to screen for TFs significantly correlated to breast cancer prognosis (*p*-value < 0.05). These prognosis-related TFs were included in the LASSO regression analysis by using the “glmnet” R package to remove redundant factors and minimize the variation in the predictive effect of the model. Next, the significant prognosis-related TFs were used to construct the prognostic risk model, and a comprehensive risk prediction model according to the contribution coefficient of each gene to the risk. Finally, in order to further visualize the influence of various factors in the risk model on prognosis, a multivariate Cox regression model was constructed and visualized using the “rms” R package.

### Evaluation of Prognosis Model and Validation of Dataset

TCGA-breast cancer data of the risk prediction model were divided into two groups according to the risk value, to test the relationship between the risk value and patient prognosis. Meanwhile, external data (METABRIC) was used to verify the model. The ROC curve and survival curve were visualized using “survivalROC” and “survival” R packages, respectively. A heat map depicting the model-related genes associated with high-risk and low-risk groups combined with the clinical status of patients was constructed using the “heatmap” R package.

### Analysis of Immune Infiltration

The CIBERSORT algorithm (http://cibersort.stanford.edu/) was employed to determine the composition of 22 types of immune infiltration-associated cells by analyzing breast cancer tissue expression profiles. CIBERSORT infers immune cell type proportions by using a signature matrix (containing 547 genes) as a reference, which represents the marker genes for each cell type *via* support vector regression. Pearson correlation analysis was used to obtain the related coefficient between the 22 immune cells. A *p*-value <0.05 was considered statistically significant.

### Construction of the TF–Target Gene Network

Target genes of differential TFs were obtained using the TRRUST transcription database (https://www.grnpedia.org/trrust/). The network graph was visualized and analyzed using Cytoscape (Version 3.6.0).

### Cell Culture

The MCF10A, MDA-MB-231, T-47D, and SUM159 cells utilized in this study were purchased from the American Type Culture Collection (Manassas, VA, USA). MCF10A cells were cultured in the base medium for this cell line (MEBM) supplemented with 100 ng/mL cholera toxin. MDA-MB-231 cells were maintained in Dulbecco’s modified Eagle’s medium (DMEM; GIBCO, Invitrogen, Grand Island, NY, USA) supplemented with 10% fetal bovine serum (FBS). T-47D and SUM159 cells were cultured in RPMI 1640 culture medium supplemented with or without bovine insulin. All the cells were maintained in a humidified incubator equilibrated with 5% CO_2_ at 37°C.

### Patients and Samples

Ten patients diagnosed with breast cancer were recruited in this study. All patients received a surgical resection at the National Cancer Center of China. The para-carcinoma and carcinoma tissues were collected immediately after surgery and stored in a preservation buffer at −80°C. Informed consent was obtained from all patients, and the use of clinical samples in this study was approved by the ethics committee of the National Cancer Center of China. The clinical characteristics of the patients are shown in **Table 3**.

**Table 3:** The clinical characteristics of breast cancer patients.

Clinical Characteristics	Age (years)	TNM	ER Status	PR Status	HER2 status	Ki67	Grade
Patient 1	67	T2N1aM0	–	–	+	50%	G3
Patient 2	63	T2N0M0	+	+	–	10%	Not applicable
Patient 3	56	T1cN2aM0	+	+	–	10%	G2
Patient 4	75	T1miN0M0	+	+	–	15%	G1
Patient 5	57	T1miN0M0	–	–	+	15%	Not applicable
Patient 6	63	T3N3M0	+	+	–	25%	G2
Patient 7	46	T2N0M0	–	–	+	50%	G3
Patient 8	32	T2N2aM0	–	–	+	70%	G3
Patient 9	54	T2N0M0	–	–	+	20%	G3
Patient 10	40	T2N3aM1	+	+	–	60%	G3

### Quantitative Real-Time PCR

Total cellular RNA was extracted from MCF10A, MDA-MB-231, T-47D, and SUM159 cells, as well as breast cancer tissues, using TRIzol reagent (Roche, Basel, Switzerland). cDNA was prepared using the Transcriptor First Strand cDNA Synthesis Kit (Roche, Basel, Switzerland). Relative quantification of select genes was determined *via* RT-PCR using the ABI PRISM 7500 System (Applied Biosystems). SYBR Green fluorescence was measured and quantified using the comparative *C*t method (2-ΔΔ*C*t) with the expression of *GAPDH* as an internal control. This assay was performed in triplicate. The primers used are listed in **Table 4**.

**Table 4:** The primers for select genes.

Gene	Forward primer	Reverse primer
*NONO*	CTAGCGGAGATTGCCAAAGTG	GTTCGTTGGACACATACTGAGG
*COPS5*	TGGGTCTGATGCTAGGAAAGG	CTATGATACCACCCGATTGCATT
*HDAC2*	ATGGCGTACAGTCAAGGAGG	TGCGGATTCTATGAGGCTTCA
*GAPDH*	GTCAACGGATTTGGTCGTAT	GAACATGTAAACCATGTAGTTGA

### Western Blotting

Sample tissues were lysed and grinded in RIPA lysis buffer (Cell Signaling Technology, USA) containing protease inhibitor cocktail (Cell Signaling Technology, USA). Total protein concentrations were determined using the Pierce™ BCA Protein Assay kit (Thermo Scientific, USA). Then, the lysed sample was mixed with an equal volume of 2× SDS-PAGE loading buffer and boiled for 10 min. The resultant materials obtained from tissues were subjected to 10% SDS-PAGE and transferred onto polyvinylidene fluoride membranes. Membranes were blocked and incubated with the appropriate antibodies (**Table 5**) overnight at 4°C, followed by incubation with secondary antibodies for 1 h at 25°C. Immunoblotting signals were detected using enhanced chemiluminescence (ECL System, Thermo Scientific, USA) according to the manufacturer’s instructions.

**Table 5:** The primary secondary antibodies for select genes.

Protein	Primary antibody	Secondary antibody
NONO	11058-1-AP (Proteintech)	5220-0336 (KPL)
COPS5	27511-1-AP (Proteintech)	5220-0336 (KPL)
HDAC2	H3159 (Sigma)	5220-0336 (KPL)
GAPDH	AC002 (ABclonal)	5220-0341 (KPL)

### Statistical Analysis

RStudio software (version 3.4.3) and GraphPad prism (version 8.0) were used to analyze the data of our study. Results are presented as the mean ± standard deviation (SD) from at least three independent experiments. Student’s *t*-test of variance was performed to compare the difference between two groups, and one-way ANOVA was used to analyze data between more than two groups. A *p-*value <0.05 was considered statistically significant.

## Data Availability Statement

The original contributions presented in the study are included in the article/**Supplementary Material**. Further inquiries can be directed to the corresponding authors.

## Ethics Statement

The studies involving human participants were reviewed and approved by the National Cancer Center of China. Written informed consent for participation was not required for this study in accordance with the national legislation and the institutional requirements.

## Author Contributions

XY were involved in the acquisition of data, prepared all figures and tables, and wrote the manuscript. JL and XW provided the human clinical samples. TY, GL, YS, XT, HY, and SW critically revised the article. WH was responsible for financial support and designed the study. All authors contributed to the article and approved the submitted version.

## Funding

This work was supported by Beijing Natural Science Foundation(7204241), the National Natural Science Foundation of China (81902960, 41931291), the State Key Laboratory of Molecular Oncology (SKLMO-KF2021-21), and Cooperative Project of Beijing-Tianjin-Hebei Regional Basic Research [19JCZDJC65700(Z)].

## Conflict of Interest

The authors declare that the research was conducted in the absence of any commercial or financial relationships that could be construed as a potential conflict of interest.

## Publisher’s Note

All claims expressed in this article are solely those of the authors and do not necessarily represent those of their affiliated organizations, or those of the publisher, the editors and the reviewers. Any product that may be evaluated in this article, or claim that may be made by its manufacturer, is not guaranteed or endorsed by the publisher.

## Supplementary Material

Supplementary Figure 1 Validation of prognostic model. **(A)** Kaplan–Meier analysis of high- and low-risk groups in breast cancer (Metabric) from the Cbioportal database. **(B)** ROC curve of 3- and 5-year survival probability in breast cancer (Metabric) from Cbioportal database.
