# Supplementary figures and images for: Identification of Key Transcription Factors and Immune Infiltration Patterns Associated With Breast Cancer Prognosis Using WGCNA and Cox Regression Analysis
Source: Front Oncol. 2021 Dec 21;11:742792. doi: 10.3389/fonc.2021.742792 (PMC8724129; doi:10.3389/fonc.2021.742792)

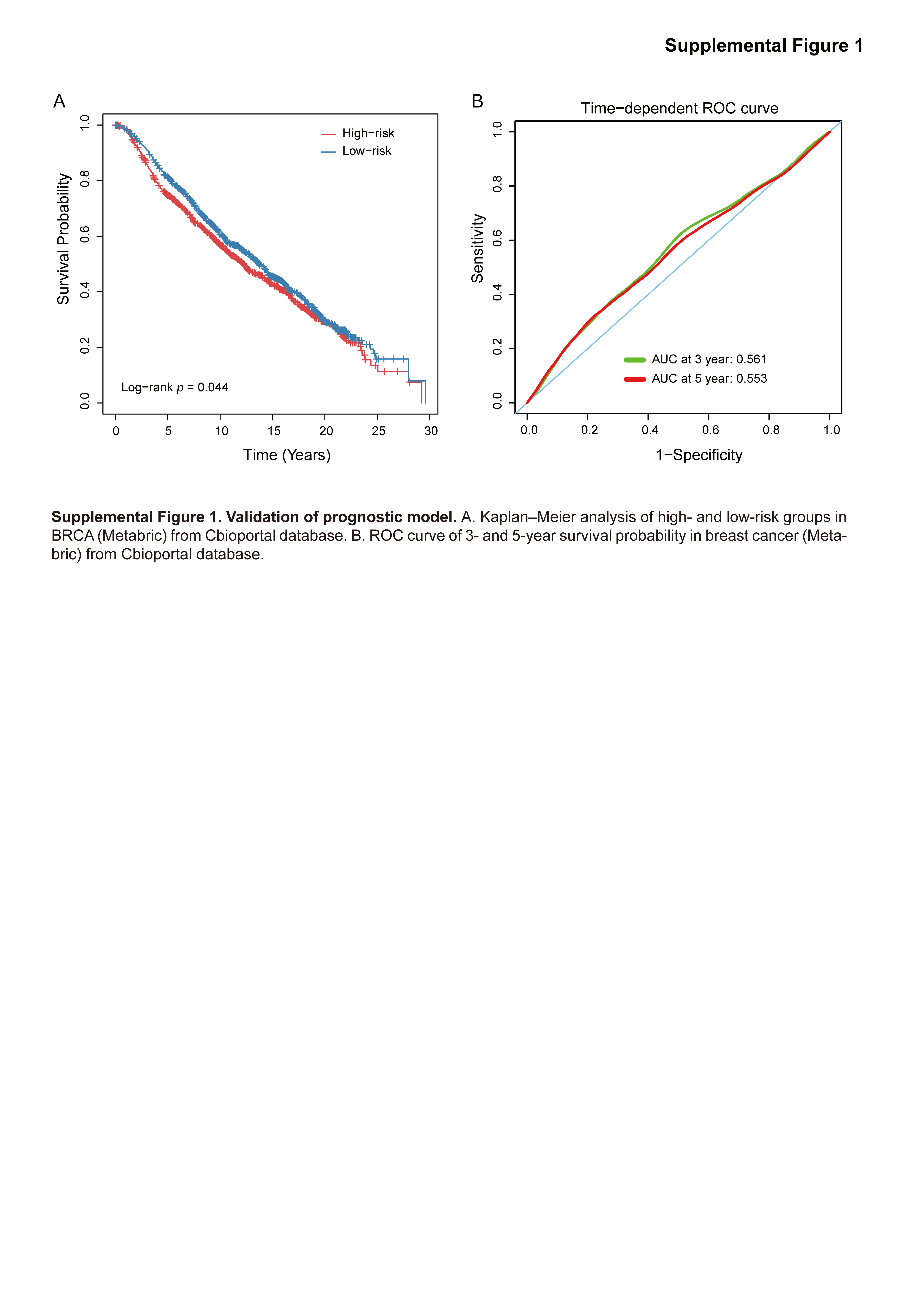

Supplement: Supplementary Figure 1 — Validation of prognostic model. (A) Kaplan–Meier analysis of high- and low-risk groups in breast cancer (Metabric) from the Cbioportal database. (B) ROC curve of 3- and 5-year survival probability in breast cancer (Metabric) from Cbioportal database. [file Image_1.tif]
